# Supplementary material for: The battle to achieve Sustainable Development Goal Two: The role of environmental sustainability and government institutions
Source: PLoS One. 2023 Sep 14;18(9):e0291310. doi: 10.1371/journal.pone.0291310 (PMC10501651; doi:10.1371/journal.pone.0291310)
Supplement: Attachment — Submitted filename: Reviewers comments.docx [file pone.0291310.s001.docx]

**Reviewer’s comments**

The study aimed to examine the role of environmental sustainability and government institutions in sustainable development goal two in West Africa.

**Introduction**

Paragraph 2: The sentence “It was estimated that the number of food-insecure people could exceed 1 billion by the end of 2020 (World Health Organization, 2020). and This number was estimated to reach 132 million by the end of 2020 (World Health Organization, 2020).” needs to be updated with recent information.

The author should focus their discussion on achieving sustainable development goal 2, and not poverty.

The introduction is lacking an important aspect, “the need for the study”. It is critical to clearly state the contribution of the study to the body of knowledge by clearly showing the research gap.

**2 Literature Review**

The author should correct the citation patterns in this heading. For instance, Similarly, the work of (Amwata et al., 2016) reveals that, in marginal areas of Kenya, households with agropastoral livelihood are less vulnerable in terms of food security as compared to their pure pastoral counterparts. (Turyahabwe et al., 2013) studies on how wetland resources contribute to household food security in agroecological zone in Uganda. There are many of these in the text.

The literature review focused on previous work without connection with the current study. it is expected that the authors will have a paragraph at the end to bring out the need for the current study to show the gaps in the literature.

The author should give the concept and theories in support of the work.

**2.1 The SDG of zero hunger**

Define SDG in the first use. Same as DG.

Correct the in-text citation. For instance, (G. L. Kennedy, 2009).

**2.2 State of Food Security in Africa**

The author should correct the citation patterns in this heading. For instance, the study by (Cooper et al., 2021) found that there is substantial diversity in the distribution of global severe food insecurity across countries and subnational regions.

**Materials**

The author should correct the citation patterns in this heading. For instance, to achieve the purpose of the study, a Generalized Method of Moments was deployed as used by (Gyimah & Yao, 2022) and Two-Staged Least Squares as used by (Nguyen & Nasir, 2021).

Data

It was stated in the abstract that the data covers 1996 to 2021, while it was stated in the methodology that it covers 1990 to 2021. The authors need to correct this.

How the data were arranged and used needs to be explained. For instance, how did the author merge the data from the 19 countries before the analysis?

The data used need to be described in terms of how WDI collected the data and so on.

Table 2 Statistical Description

The results here show that the data are logged. Clearly state if the data are logged or not.

Table 3 Correlation

There are some variables with high correlation. How did the authors take care of these variables?

**Results and Discussions**

The results of the findings were not well discussed. The author should explain each of the significant variables, state the reasons behind the results and relate them to the literature. For instance, how did carbon emissions positively affect the food production index, agriculture, forestry and fishing, and forest area? This and other results need to be critically explained for readers to understand.

Considering that the countries in West Africa have different policies, Governance institutions, Renewable energy consumption and population growth, among others. how did the authors take care of these differences? In addition, a specific investigation and analysis for each country selected would be better. This will enable us to understand what affects food insecurity in each nation.

**Conclusion and policy implication**

The conclusion should be concise; it is not a summary of the work. Policy implications should be directly from the findings of the study.
