# Supplementary material for: The battle to achieve Sustainable Development Goal Two: The role of environmental sustainability and government institutions
Source: PLoS One. 2023 Sep 14;18(9):e0291310. doi: 10.1371/journal.pone.0291310 (PMC10501651; doi:10.1371/journal.pone.0291310)
Supplement: Attachment — Submitted filename: REVIEWERS COMMENT-PONE.docx [file pone.0291310.s002.docx]

**REVIEWERS COMMENTS**

**Summary**

Food security remains one of the top priorities of the United Nations Sustainable Development Goals (SDG), which aims to alleviate hunger (zero Hunger) by 2030. Eliminating food insecurity, hunger and malnutrition by 2025 has been the main target of the African Union's Agenda 2063. Achieving this goal comes with its own challenges considering the need to feed more people from the expected increasing global population coupled with the impacts of climatic changes on food production. Population growth has brought a high demand for limited world resources, most notably food supplies. In the era of globalization coupled with the projected increase in world population, the need to attain global food security and poverty reduction becomes a global challenge for humanity in achieving sustainability. Population density and climate change threaten the second sustainable development goal (zero hunger); attaining food and its related environmental security comes with significant challenges. The author, therefore, explores the current state of food security within sub-Saharan Africa and the connection of food security with various contributing factors such as economic growth, governance, geopolitical, climatic, population, and environmental factors and the effect of environmental sustainability and government institutions on food security in West Africa with data from 1996 to 2021, two models are employed using the Generalized Method of Moments as the primary model and Two-Stage Least Squares for the robustness check. Findings from this study revealed that carbon emissions representing environmental sustainability have no direct significant effect on food security, while government institutions reduce food security. Also, income and urbanization promote food security, while renewable energy and population growth reduce food security.

**Major comments**

The methods, results, and discussion are well documented with good clarity and are scientific.

**Minor comments**

1. In the abstract, the authors should do some grammar checks on some tenses (appropriate use of past tense).

2. The references should be improved upon and should be consistent across the manuscript. In some cases Reference were omitted. Intext references, where the author’s initial appears should be corrected appropriately. e.g undernourished (P. Sharma et al., 2016).

Also, sentences like “the study by (Frelat et al., 2016) reveals that over 30% of rural households across 17 countries within the sub–Saharan Africa are still struggling with food insecurity” where brackets enclose author’s name within a statement, such brackets should enclose only the year e.g Frelat et al. (2016). This correction should be effected across the whole manuscript.

In their study, it was observed that households go through a transitional phase of inadequate food security during planting and growing seasons to fully secured food security during periods of harvesting.

This statement “The increase in population growth certainly has a connection to food security, ranging from the changes in human diets to the methods and ways through which food is produced. For instance, the increase in population will also mean more people are becoming wealthier and thus will be able to eat more and regularly or more people turn to opt for more resource-intensive food, thereby leading to the decline of resources needed for food production” has no reference. (page 13, paragraph 2)

This statement “The proper utilization of food could help address issues of food allocation, as well as the dietary quality of food. Given the multidisciplinary nature of food security, many disciplines have engaged in studies of food security issues and how these disciplines and sectors influence food security, including agriculture, anthropology, economics, nutrition, public policy, and sociology, as have numerous national and international governmental and non-governmental agencies”. Has no reference (page 15, paragraph 1).

Last paragraph on page 17 has no reference- “Also, one key element of Africa's food security is the unavailability of food from either domestic production or the inability to access the global market, accessibility of food by consumers, affordability, and safety”

The last paragraph on page 19, in the literature review section, just before the methodology has no reference, here “Food insecurity remains a major challenge in Africa, and it is estimated that nearly 250 million people are still at risk of hunger and malnutrition. In addition, climate change is expected to worsen the situation, with increased droughts, floods, and other extreme weather events projected to increase food insecurity in the region”.

Paragraph 1 of the result and discussion section on page 22 has no reference “These institutions are expected to provide and implement policies that will promote food sustainability however since West Africa over the past years has faced institutional changes especially political and economic governance, these institutions are not strong enough to promote food security in the region”.

Similarly, most information in the result and discussion section was not properly referenced.

“Carbon emissions has positive and significant effect on government institutions and government institutions have the same effect on carbon emissions in the second dimension of the results. This indicates that they influence each other in the same direction. Income level and urbanization improve government institutions, while renewable energy and population growth have negative significant effect on government institutions”. Can this statement be compared with previous studies?

**Conclusion/recommendation**

Generally, the study titled was well-researched, very relevant, scientific, and very informative, it raised pertinent issues regarding the impact of government institutions and environmental sustainability on achieving the “zero hunger” which is the Sustainable Development Goal 2.

The authors demonstrated excellent mastery of the study. However, few minor corrections are required as highlighted.
