# Supplementary material for: The battle to achieve Sustainable Development Goal Two: The role of environmental sustainability and government institutions
Source: PLoS One. 2023 Sep 14;18(9):e0291310. doi: 10.1371/journal.pone.0291310 (PMC10501651; doi:10.1371/journal.pone.0291310)
Supplement: Attachment — Submitted filename: Response to Reviewers.docx [file pone.0291310.s003.docx]

**Response to Reviewers’ Comments**

**Reviewer #1**

**Comment:** Paragraph 2: The sentence “It was estimated that the number of food-insecure people could exceed 1 billion by the end of 2020 (World Health Organization, 2020). and This number was estimated to reach 132 million by the end of 2020 (World Health Organization, 2020).” needs to be updated with recent information.

**Response:** Thank you for the comment. The paragraph has been revised and updated

**Comment:** The author should focus their discussion on achieving sustainable development goal 2, and not poverty.

**Response:** Thank you for pointing this out. The manuscript has been revised accordingly

**Comment:** The introduction is lacking an important aspect, “the need for the study”. It is critical to clearly state the contribution of the study to the body of knowledge by clearly showing the research gap.

**Response:** Thank you for the comment. The introduction has been revised accordingly

**Comment:** The author should correct the citation patterns in this heading.

**Response:** Thank you for the comment. The citations in the manuscript has been corrected accordingly

**Comment:** The literature review focused on previous work without connection with the current study. it is expected that the authors will have a paragraph at the end to bring out the need for the current study to show the gaps in the literature.

**Response:** Thank you for the comment. The literature review has been revised and updated to reflect the objective and aim of the current study

**Comment:** The author should give the concept and theories in support of the work

**Response:** Thank you for the comment. The concept and theories of the study has been provided to support the study

**Comment:** It was stated in the abstract that the data covers 1996 to 2021, while it was stated in the methodology that it covers 1990 to 2021. The authors need to correct this.

**Response:** Thank you for pointing this out. The authors have corrected mistake.

**Comment:** How the data were arranged and used needs to be explained. For instance, how did the author merge the data from the 19 countries before the analysis?

**Response:** Thank you for the comment. The data used for the study is panel data and the authors used Excel to merge the data.

**Comment:** The data used need to be described in terms of how WDI collected the data and so on.

**Response:** Thank you for the comment. The authors have described the measurement of the variables and incorporated them in Table 1.

**Comment:** The results here show that the data are logged. Clearly state if the data are logged or not.

**Response:** Thank you for the comment. The authors have stated the form of the statistical descriptive data in the data section (logged form)

**Comment:** There are some variables with high correlation. How did the authors take care of these variables?

**Response:** Thank you for the comment. The authors did preliminary tests (cross-section dependency test, panel unit root test, and panel cointegration test) to check the suitability of the variables and the results revealed that the variables are suitable for the study.

**Comment:** The results of the findings were not well discussed. The author should explain each of the significant variables, state the reasons behind the results and relate them to the literature. For instance, how did carbon emissions positively affect the food production index, agriculture, forestry and fishing, and forest area? This and other results need to be critically explained for readers to understand.

**Response:** Thank you for pointing this out. The authors have explained and backed their findings with literature as suggested

**Comment:** Considering that the countries in West Africa have different policies, Governance institutions, Renewable energy consumption and population growth, among others. how did the authors take care of these differences? In addition, a specific investigation and analysis for each country selected would be better. This will enable us to understand what affects food insecurity in each nation.

**Response:** Thank you for the comment. The authors did preliminary tests to examine if the variables are well presented for the study. The tests show that the variables are well represented and suitable for panel study. In addition, the study did not include country base result because of the objective of the study thus to assess regional effect.

**Comment:** The conclusion should be concise; it is not a summary of the work. Policy implications should be directly from the findings of the study.

**Response:** Thank you for the comment. The conclusion and policy implications has been revised accordingly to reflect the findings of the study

**Reviewer #2**

**Comment:** In the abstract, the authors should do some grammar checks on some tenses (appropriate use of past tense).

**Response:** Thank you for the comment. Grammar checks and all necessary corrections has been carried out.

**Comment:** The references should be improved upon and should be consistent across the manuscript

**Response:** Thank you for the comment. Citations in the entire manuscript has been corrected accordingly and necessary reference has been provided to support the claims made.

**Comment:** Are there other findings that support or contradict this assertion?

**Response:** Thank you for the comment. The section has been revised.

**Comment:** Any contrary/similar opinions from previous findings with references?

**Response:** Thank you for the comment. The authors have addressed this issue and relevant findings have been cited.
