# Supplementary material for: The battle to achieve Sustainable Development Goal Two: The role of environmental sustainability and government institutions
Source: PLoS One. 2023 Sep 14;18(9):e0291310. doi: 10.1371/journal.pone.0291310 (PMC10501651; doi:10.1371/journal.pone.0291310)
Supplement: Attachment — Submitted filename: PLOSONE FINAL COMMENT OF ACCEPTANCE 16082023.docx [file pone.0291310.s004.docx]

**Reviewers’ final assessment.**

The author has satisfactorily addressed the areas of concern as stipulated in the reviewer’s comment.
